# Supplementary material for: European Stroke Organisation (ESO) and European Society for Minimally Invasive Neurological Therapy (ESMINT) guideline on acute management of basilar artery occlusion
Source: J Neurointerv Surg. 2024 Jul 22;16(9):e7. doi: 10.1136/jnis-2024-022053 (PMC11347260; doi:10.1136/jnis-2024-022053)
Supplement: Supplementary data [file jnis-2024-022053supp001.pdf]

Supplemental Table 1. Conflicts of interest of module working group members

Author	Discipline and affiliation	Intellectual and financial disclosures
Daniel Strbian	Department of Neurology<br>HUS Neurocenter, Helsinki University Hospital and University of Helsinki, Helsinki, Finland	Intellectual disclosures:<br>-European Stroke Organization (ESO): Executive committee<br>-Member of the ESO Guideline board<br>-National Coordinator of SITS registry<br>-Assistant Editor “Stroke” Journal<br>-Co-author of ESO Guideline on IVT for AIS (PMID: 33817340)<br>-Co-author of ESO Guideline on IVT before MT in AIS pts with LVO (PMID: 35342811)<br>-Steering committee of RCTs: DISTAL, PROOF, SWIFT-DIRECT, ELAN, SWITCH, Milvexian SSP, Librexia, TECNO, ICARUS<br>Financial disclosures:<br>-Advisory Board: Astra-Zeneca, Alexion, CSL Behring, Shionogi, BMS, Janssen<br>-Unrestricted Research or Educational Grants: Boehringer-Ingelheim
Caroline Arquizan	Department of Neurology, Stroke Unit, Montpellier University Hospital	Steering commitee of RCT In EXTREMIS (MOSTE and LASTE) which was funded by Montpellier University Hospital through an unrestricted grant from an industry consortium (MEDTRONIC, STRYKER, BALT EXTRUSION, MICROVENTION, CERENOVUS)<br>Intellectual disclosure : General Secretary of the Societe Française NeuroVascualire<br>Personal honoraria from speaker honoraria from MEDTRONIC, AMGEN
Petra Cimflova	MD, PhD, Radiologist<br>Department of Radiology, University of Calgary, Calgary, Canada<br>Department of Medical Imaging, St. Anne’s University Hospital Brno and Faculty of Medicine MU Brno, Brno, Czech Republic<br>Klinik für Neuroradiologie, Universitaetsklinikum Freiburg, Breisacher Strasse 64, 79106 Freiburg, Germany	None

Jens Fiehler	Neuroradiology, UMC Hamburg-Eppendorf	Consultant for Acandis, Cerenovus, Medtronic, Microvention, Penumbra, Phenox, Roche, Stryker. Stocks of Tegus, Eppdata and Vastrax.
Georgios Georgiopoulos	MD, PhD, Cardiologist<br>Department of Physiology, School of Medicine, University of Patras, Grece and School of Biomedical Engineering and Imaging Sciences, King's College London, UK	None
Jan Gralla	MD, MSC<br>Department of Diagnostic and Interventional Neuroradiology, Inselspital University of Bern, Switzerland	Global PI of STAR (NCT01327989) and Swift Direct (NCT03192332) (Medtronic), Consultancy<br>Consultancy for Johnson & Johnson/Cerenovus
Patrik Michel	Neurology Service, Department of Clinical Neurosciences, Lausanne University Hospital and University of Lausanne, Lausanne, Switzerland	No funding related to this project.<br>Unrelated: Research support to my institution from the Swiss National Science Foundation, the Swiss Heart Foundation, and Faculty of Biology and Medicine of the Lausanne University<br>Intellectual: member of the Steering committee, and local PI of the BASICS trial
Johanna Ospel	Neuroradiology, Department of Diagnostic Imaging, Foothills Medical Centre, University of Calgary, Alberta, Canada	Consultant to Nicolab (unrelated to the guidelines)
Silja Räty	Neurology, Department of Neurology, Helsinki University Hospital, Finland	None
Georgios Tsivgoulis	Second Department of Neurology, "Attikon" University Hospital, School of Medicine, National and Kapodistrian University of Athens, Athens, Greece	Intellectual disclosures:<br>-European Stroke Organization (ESO): Vice President<br>-Hellenic Neurological Society: President

		-Hellenic Society of Cerebrovascular Diseases: General Secretary<br>-Member of the ESO Guideline board<br>-Chair of ESO Industry Roundtable<br>-National Coordinator of SITS, ANGELS, RES-Q registries<br>-Member of SITS Scientific Committee<br>-Section Editor “Stroke” Journal<br>-Associate Editor: Therapeutic Advances in Neurological Disorders, Journal of Neuroimaging<br>-Co-author of ESO Guideline on IVT for AIS (PMID: 33817340)<br>-Co-author of ESO Guideline on IVT before MT in AIS pts with LVO (PMID: 35342811)<br>-Co-author of ESO Guideline on Tenecteplase for patients with acute ischaemic stroke (doi: 10.1177/23969873221150022)<br>-Adjudication committee of BI 1123-0040 RCT (Phase 3 trial evaluating safety and efficacy of TNK vs. TPA in AIS)<br>-Steering committee of DISTAL RCT (Phase 3 trial evaluating the safety and efficacy of EVT in DMVOs)<br>Financial disclosures:<br>-Participation in Advisory Meetings & Satellite Symposia: Novartis, Sanofi, Biogen, Genesis Pharma, Teva, Shire, Merck, Bayer, Daichii-Sankyo, Allergan, Specifar, Actavis, Boehringer-Ingelheim, Medtronic, CSL Behring, Abbott, Takeda, Abbvie, Ipsen, ITF, Shionogi, Novasignal, BMS, Roche, Medison, Astra<br>-Unrestricted Research or Educational Grants: Novartis, Genesis Pharma, Teva, Shire, Merck, Abbott, Allergan, Boehringer-Ingelheim, Medtronic, Amicus, Abbvie, Ipsen, Bayer, Roche, Novalis
Guillaume Turc	GHU Paris Psychiatrie et Neurosciences, 1 rue Cabanis, 75014 Paris, France	Lecture fees: Guerbet France

Salman Hussain	European Stroke Organisation, Basel, Switzerland	None
Teresa Ullberg	M.D, PhD, Neurology, Department of Clinical Sciences Lund, Lund University, Skane University Hospital, Lund and Malmö, Sweden	TU received personal honoraria from an Expert assignment for Astra Zeneca, and speaker honoraria from Siemens Healthineers.
Kamil Zeleňák	Clinic of Radiology, Jessenius Faculty of Medicine, Comenius University, Kollárova 2, 03659 Martin, Slovakia.	Steering committee of the TENSION study - TENSION has received funding from the European Union’s Horizon 2020 research and innovation programme under grant agreement No 754640.
Wim van Zwam	Department of Radiology and Nuclear Medicine , Maastricht University Medical Center<br>Maastricht, The Netherlands	Co P.I. of MrClean and MrClean-Late studies, which received funding from the Dutch Heart Foundation, Health Holland, Dutch Brain Council, Medtronic, Penumbra, Stryker and Cerenovus.<br>Speaker fees from: Stryker, Cerenovus, NicoLab and Philips, all paid to institution.<br>DSMB chair: WeTrust, InExtremis, ANAIS

Supplemental Table 2. List and rating (mean score) of the selected outcomes for each PICO question.

Outcome / PICO	1	2	3	4	5	6	7	8	9	10
mRS 0-3 at 3 months	7.7	7.8	7.8	7.4	7.2	6.9	7.5	7.0	7.4	7.4
mRS 0-2 at 3 months	8.0	7.5	7.5	7.1	7.0	6.7	7.0	6.7	6.9	6.9
shift mRS at 3 months	8.4	8.4	8.4	8.0	7.9	7.6	8.1	7.7	7.9	7.9
mortality at 3 months	6.9	7.2	7.2	7.0	7.0	6.7	6.9	6.4	6.9	7.1
sICH	6.4	6.2	6.2	5.6	5.7	5.6	6.5	6.1	6.4	7.1
mTICI 2B/3	4.8	5.2	5.2	4.7	4.9	4.8	5.3	6.2	6.8	6.1

Supplemental Table 3. Literature search

Ovid MEDLINE and Embase(R) ALL <1946 to January 13, 2023>

#	search string
**1**	exp Basilar Artery/
**2**	basilar.ti,ab,kw.
**3**	exp Arterial Occlusive Diseases/
**4**	'basilar artery occlusion'.mp.
**5**	'basilar artery obstruction'.mp.
**6**	'acute basilar artery occlusion'.mp.
**7**	artery occlusion.mp.
**8**	blood vessel occlusion.mp.
**9**	BAO.mp.
**10**	Occlusion.mp.
**11**	Occlusions.mp.
**12**	'basilar artery occlusions'.mp.
**13**	Vertebrobasilar Insufficiency/
**14**	vertebrobasilar occlusion.mp.
**15**	vertebrobasilar.mp.
**16**	vertebrobasilar circulation.mp.
**17**	posterior circulation.mp.
**18**	'posterior cerebral'.mp.
**19**	vertebral.mp.
**20**	"pc-ASPECTS ".mp.
**21**	"Posterior Circulation ASPECTS".mp.

**22**	'Basilar Artery International Cooperation Study'.mp.
**23**	or/1-22
**24**	cerebrovascular accident.mp.
**25**	cerebrovascular disorders/ or basal ganglia cerebrovascular disease/ or exp brain ischemia/ or carotid artery diseases/ or carotid artery thrombosis/ or intracranial arterial diseases/ or cerebral arterial diseases/ or exp "intracranial embolism and thrombosis"/ or exp stroke/
**26**	(isch?emi$ adj6 (stroke$ or apoplex$ or cerebral vasc$ or cerebrovasc$ or cva)).tw.
**27**	((brain or cerebr$ or cerebell$ or vertebrobasil$ or hemispher$ or intracran$ or intracerebral or infratentorial or supratentorial or middle cerebr$ or mca$ or anterior circulation) adj5 (isch?emi$ or infarct$ or thrombo$ or emboli$ or occlus$ or hypoxi$)).tw.
**28**	((brain$ or cerebr$ or cerebell$ or intracerebral or intracran$ or parenchymal or intraparenchymal or intraventricular or infratentorial or supratentorial or basal gangli$ or putaminal or putamen or posterior fossa or hemispher$ or subarachnoid) adj5 (h?emorrhag$ or h?ematoma$ or bleed$)).tw.
**29**	or/24-28
**30**	23 and 29
**31**	radiography, interventional/ or radiology, interventional/
**32**	catheterization/ or angioplasty/ or angioplasty, balloon/ or angioplasty, balloon, laser-assisted/ or angioplasty, laser/ or atherectomy/ or catheter ablation/
**33**	Stents/
**34**	mechanical thrombolysis/ or thrombectomy/ or embolectomy/
**35**	endovascular thrombectomy.mp.
**36**	endovascular therapy.mp.
**37**	endovascular treatment.mp.
**38**	'NIHSS score'.mp.
**39**	blood vessel prosthesis/ or blood vessel prosthesis implantation/

**40**	cerebral revascularization/ or reperfusion/ or dilatation/
**41**	(interventional adj3 (radiolog$ or radiograph$ or neuroradiolog$)).tw.
**42**	(angioplast$ or stent$).tw.
**43**	(thrombectomy or embolectomy or atherect$).tw.
**44**	(thromboaspiration or arterial recanali?ation).tw.
**45**	((mechanical or radiolog$ or pharmacomechanical or laser or endovascular or neurovascular) adj5 (thrombolys$ or reperfusion or fragmentation or aspiration or recanali?ation or clot lys$)).tw.
**46**	((clot or thrombus or thrombi or embol$) adj5 (aspirat$ or remov$ or retriev$ or fragment$ or retract$ or extract$ or obliterat$ or dispers$ or disrupt$ or disintegrate$)).tw.
**47**	((retrieval or extraction) adj5 device$).tw.
**48**	endoluminal repair$.tw.
**49**	((merci or concentric) adj retriever).tw.
**50**	(endovascular snare$ or neuronet or microsnare or X-ciser or angiojet).tw.
**51**	thrombolytic therapy/
**52**	fibrinolytic agents/ or fibrinolysin/ or plasminogen/ or tissue plasminogen activator/ or exp plasminogen activators/ or urokinase-type plasminogen activator/ or exp streptokinase/
**53**	fibrinolysis/
**54**	(thromboly$ or fibrinoly$ or recanalis$ or recanaliz$).tw.
**55**	((clot$ or thrombus) adj5 (lyse or lysis or dissolve$ or dissolution or bust$)).tw.
**56**	(tPA or t-PA or rtPA or rt-PA or plasminogen or plasmin or alteplase or actilyse).tw.
**57**	(tPA or t-PA or rtPA or rt-PA or plasminogen or plasmin or alteplase or actilyse).nm.
**58**	(anistreplase or streptodornase or streptokinase or urokinase or pro?urokinase or rpro?uk or lumbrokinase or duteplase or lanoteplase or pamiteplase or reteplase or saruplase or staphylokinase or streptase or tenecteplase or desmoteplase or amediplase or monteplase or nasaruplase or silteplase).tw.

**59**	(anistreplase or streptodornase or streptokinase or urokinase or pro?urokinase or rpro?uk or lumbrokinase or duteplase or lanoteplase or pamiteplase or reteplase or saruplase or staphylokinase or streptase or tenecteplase or desmoteplase or amediplase or monteplase or nasaruplase or silteplase).nm.
**60**	or/31-59
**61**	Epidemiologic Studies/
**62**	exp Case Control Studies/
**63**	exp Cohort Studies/
**64**	(epidemiologic adj (study or studies)).ab,ti.
**65**	case control.ab,ti.
**66**	(cohort adj (study or studies)).ab,ti.
**67**	cohort analy$.ab,ti.
**68**	(follow up adj (study or studies)).ab,ti.
**69**	longitudinal.ab,ti.
**70**	retrospective$.ab,ti.
**71**	prospective$.ab,ti.
**72**	(observ$ adj3 (study or studies)).ab,ti.
**73**	registry study.mp.
**74**	randomised controlled trial.pt.
**75**	controlled clinical trial.pt.
**76**	randomised.ab.
**77**	placebo.ab.
**78**	clinical trials as topic.sh.
**79**	randomly.ab.
**80**	trial.ti.

**81**	or/61-80
**82**	30 and 60 and 81
**83**	exp animals/ not humans.sh.
**84**	82 not 83

Final hits: 11766
